# Editorial: (Pushing) the Limits of Neuroplasticity Induced by Adult Language Acquisition

**DOI:** 10.3389/fpsyg.2018.01806

**Published:** 2018-09-25

**Authors:** Jurriaan Witteman, Yiya Chen, Leticia Pablos-Robles, Maria Carmen Parafita Couto, Patrick C. M. Wong, Niels O. Schiller

**Affiliations:** ^1^Department of Linguistics, Leiden University, Leiden, Netherlands; ^2^Leiden University Centre for Linguistics, Leiden, Netherlands; ^3^Leiden Institute for Brain and Cognition, Leiden University, Leiden, Netherlands; ^4^Department of Linguistics and Modern Languages, Brain and Mind Institute, The Chinese University of Hong Kong, Hong Kong, China

**Keywords:** brain, cognition, language, bilingualism, neuroplasicity

Many individuals attempt to learn a second (L2) or even third language (L3) at some point in their life. Since language exposure is one of the most intense cognitive training regimes one can encounter, it is not surprising that previous research has shown that multilingualism can induce profound neural changes or “neuroplasticity” (Costa and Sebastián-Gallés, 2014). Despite the general consensus that learning a new language in adulthood can change the brain, what remains unclear is the *scope* of such neuroplasticity. In other words, what limits vs. promotes neurocognitive change as a result of second language acquisition in adulthood?

On the one hand, there are factors that may limit such change of the neurocognitive system due to L2 (or L3) acquisition. For instance, models of adult L2 learning assume that acquisition of the mother tongue (L1) has sculpted neural circuits to discriminate between L1 linguistic elements which in turn limits the ability to distinguish between L2 elements (e.g., van Leussen and Escudero, 2015). On the other hand, there might be factors that enhance L2 induced neurocognitive change, such as language aptitude (Hu et al., 2013; Chai et al., 2016) and the intensity (Tremblay et al., 1997; Thomson and Derwing, 2015) and quality (Zhang et al., 2009; Ylinen et al., 2010; Morgan-Short et al., 2012; Grimaldi et al., 2014) of the L2 acquisition regime. Hence, much is yet to be investigated about the factors that limit vs. promote adult language learning induced neuroplasticity as well as the mediating underlying neurocognitive mechanisms. The present research topic therefore aimed to identify some of the factors that limit or promote adult L2 learning induced neurocognitive plasticity and the underlying neurocognitive mechanisms.

What factors then, might limit neurocognitive change due to adult L2 acquisition? The two reviews presented in the current research topic (Birdsong; Antoniou and Wright) both suggest that having reached adult age itself might be a limiting factor because adult age represents a period of relatively (as compared to childhood) low susceptibility to L2 exposure, limiting the degree to which L2 proficiency can be gained. Additionally, the mismatch of L1-L2 typology was suggested to limit L2 acquisition, with a relatively large mismatch delaying successful L2 acquisition (Antoniou and Wright). Indeed, a cross-linguistic priming study with concurrent ERP recordings presented in the current research topic showed that already early in the L2 acquisition process there is interaction between L1 and L2 at the semantic level (Meade et al.). Another study (Yang et al.), showed that a large typological difference between L1 and an L3 makes switching between languages in bilinguals more difficult with different underlying cognitive control networks being engaged in switching between balanced vs. unbalanced languages.

Acquisition of L2 (and underlying neurocognitive change) may additionally vary *between* domains of the language or even *within* a domain. Indeed, a study presented in the current research topic showed that while learning an artificial language, words that correspond to relatively concrete concepts are more easily integrated into existing semantic networks than words that refer to relatively abstract concepts (Ding et al.).

Finally, as outlined above (adult) age may itself limit neuroplastic change due to L2 learning. Indeed, a structural white matter imaging study presented in the current research topic suggests that white matter bundles critical for obtaining syntactic abilities are still developing in adolescence but may have reached maturation in adults (Yamamoto and Sakai), perhaps limiting acquisition of L2 syntax at adult age. On the other hand, the limiting effect of age of acquisition on L2 induced structural neuroplasticity may in itself be limited, as shown by a study presented in the current research topic, demonstrating white matter differences purportedly due to L2 learning between mono- and bilinguals, despite of L2 learners having reached adulthood (Rossi et al.).

Having discussed the factors that may limit neuroplasticity due to adult L2 acquisition, what factors may promote it? One of the reviews of the present research topic (Birdsong) mentions some possible factors such as: high working memory capacity, motivation to learn and meta-linguistic awareness (which could be promoted by having successfully acquired a previous non-native language). Indeed, a study in the present research topic examining predictors of L2 acquisition success found evidence that high working memory capacity predicts L2 acquisition success (Blumenfeld et al.). Furthermore, the general ability to learn or “language aptitude” may enhance neurocognitive change induced by L2 or L3 learning. Indeed, a study presented in our research topic investigating the morphology of Heschl's gyrus (HG), the primary auditory cortex, suggests that the number of complete duplications of HG in the right hemisphere might be a structural correlate of language aptitude, that may enhance L2 acquisition success (Turker et al.). Finally, in an interesting study examining the effects of L2 acquisition on *L1* processing presented in the current research topic (Kasparian and Steinhauer), very extended exposure to L2 and resulting high L2 proficiency emerged as an important factor in determining (abnormal) morphosyntactic L1 processing, suggesting that the intensity of L2 exposure is a critical determinant of neuroplastic change in the underlying neurocognitive architecture of the language processing system.

In sum, the studies presented in the current research topic suggest that neuroplastic change due to acquisition of another language (L2, L3, etc.) seems to be limited by adult age, typological mismatch between the already acquired and to be acquired languages, and limited exposure to the to be acquired language. On the other hand, high working memory capacity, high “language aptitude,” and a high level of exposure to the to be acquired language seem to promote neuroplastic change. Together, we have aimed with the studies presented in the current research topic to provide a fresh look at the scope of neuroplastic change due to adult second language acquisition.

## Author contributions

Conceived the idea: JW and NS; Wrote the Research Topic proposal: JW, YC, LP-R, MP, PW, and NS; Edited articles: JW, YC, LP-R, MP, PW, and NS; Wrote the editorial: JW.

### Conflict of interest statement

PW is co-owner of a tech startup company in Hong Kong that is related to this research topic. The remaining authors declare that the research was conducted in the absence of any commercial or financial relationships that could be construed as a potential conflict of interest.
